# SULT2B1 promotes cholangiocyte epithelial-mesenchymal transition in biliary atresia: one baby step or a giant leap in the pathogenesis of biliary atresia?

**DOI:** 10.1038/s41390-025-04688-5

**Published:** 2026-01-20

**Authors:** Rosie E. Balfour-Lynn, Anil Dhawan

**Affiliations:** Paediatric Liver, GI and Nutrition Centre and MowatLabs, King’s College Hospital, London, UK

## Abstract

Biliary atresia (BA) is a progressive fibro-inflammatory cholangiopathy and is the most common indication for paediatric liver transplantation. Its pathogenesis remains elusive, and Kasai portoenterostomy is still the mainstay of management, with no current disease-modifying medical treatments available. The study by Yang et al. aims to further elucidate the pathogenesis of BA by exploring mechanisms of epithelial-mesenchymal transition (EMT) in BA, finding evidence that SULT2B1 promotes EMT via the Wnt/β-catenin pathway and MMP7. SULT2B1 was found to be overexpressed in BA livers, and increased expression was associated with the severity of fibrosis and poor prognosis. There was evidence that SULT2B1 expression was correlated with MMP7-mediated epithelial-mesenchymal cholangiocyte transition. In vitro studies showed that TGF-β1 treatment led to overexpression of SULT2B1 and promoted the EMT process via the Wnt/β-catenin/MMP7 pathway, while silencing of SULT2B1 reduced this. These findings represent a step forward in our understanding of the pathogenesis of BA, but further work is needed to translate this to clinical practice. The work of Yang et al. suggests multiple possible routes for this, including further investigation of the potential for SULT2B1 blockade, use of MMP7 as a prognostic marker or as a therapeutic target, and investigation of the relationship between SULT2B1 and CMV.

Biliary atresia (BA) is a progressive fibro-inflammatory cholangiopathy of the intra- and extrahepatic bile ducts. Its pathogenesis remains elusive, and issues in the developmental process, environmental triggers such as viral infection, immune dysregulation and genetic susceptibility may all be involved to varying degrees.^1,2^ BA remains the most common indication for paediatric liver transplantation. Kasai portoenterostomy remains the mainstay of management, with no disease-modifying medical treatments currently available.

Epithelial-mesenchymal transition (EMT) is a process by which epithelial cells lose epithelial characteristics and transition into mesenchymal cells.^3^ EMT was first described in the early 1980s,^4^ and has been implicated in liver fibrosis.^5^ However, its significance in BA remains uncertain.^6,7^

In their study in this issue of Pediatric Research, Yang et al. began by identifying key molecules involved in the EMT process in BA.^8^ Weighted gene co-expression network analysis was performed on a publicly available RNA-Seq dataset comprising 171 BA liver tissue samples and 7 normal controls. One module associated with lower native liver survival was enriched in signalling pathways involved in EMT. Comparison of high- and low-native liver survival groups within this module identified differentially expressed genes (DEGs), of which sulfotransferase family 2b member 1 (SULT2B1) was the highest-fold change DEG. SULT2B1 is an enzyme involved in the synthesis of cholesterol sulphates. Previous studies show involvement in angiogenesis in gastric cancer^9^ and in proliferation and invasion of colorectal cancer cells.^10^ Work in primary mouse hepatocytes has suggested SULT2B1 may be involved in EMT, but it is previously unknown whether this is the case in BA.^11^

Matrix metalloproteinase 7 (MMP7) was also a significantly upregulated DEG. MMP7 is an enzyme which degrades extracellular matrix.^6^ It has been suggested as a biomarker for diagnosis of BA^12–14^ and is associated with the severity of fibrosis in multiple studies.^15–20^ Although there is much circumstantial evidence for the involvement of MMP7 in the pathophysiology of BA, the mechanism for this is unclear. It has previously been hypothesised that MMP7 could activate the Wnt/β-catenin pathway, which could be the mechanism for its involvement in the progression of fibrosis.^6^

The Wnt/β-catenin pathway is involved in the determination of cell fate and proliferation of cells,^21^ and is one of the signalling pathways known to be involved in initiating and driving EMT.^3^ A previous study has shown upregulation of Wnt/β-catenin signalling proteins in BA livers compared to normal controls, and protein expression of the products of the Wnt/β-catenin pathway was correlated to hepatic fibrosis, suggesting it has a role in the progression of fibrosis in BA.^22^ Yang et al. performed further gene set enrichment analysis on the RNA-Seq dataset, which showed that high expression of SULT2B1 appeared to be significantly involved with EMT and Wnt/β-catenin pathways. There was a significant trend for SULT2B1/MMP7 co-expression, and both were positively correlated with EMT and fibrosis-related genes, suggesting they are involved in EMT in BA. As a result of this, SULT2B1 and MMP7 were chosen for further investigation.

RNA-Seq techniques were then used on liver tissue samples obtained from 31 patients with BA at Kasai and 20 ‘healthy controls’ (normal adjacent non-tumour tissue from hepatoblastoma patients with normal biopsy findings). There was a correlation between SULT2B1/MMP7 gene expression and mesenchymal-associated gene expression. There was also a correlation with clinical findings: SULT2B1/MMP7 expression and liver stiffness on elastography were positively correlated, and SULT2B1 was upregulated in the group with ongoing jaundice at 6 months old. Western blotting confirmed that SULT2B1 and MMP7 proteins, as well as mRNA, were overexpressed in BA livers compared to healthy controls. Mesenchymal protein markers, including N-cadherin, were also increased in BA livers, as was β-catenin. Using immunofluorescence and immunohistochemistry techniques, SULT2B1 and MMP7 expression were localised primarily to cholangiocytes.

Under pathological conditions, cells, including pro-apoptotic and inflammatory cells, particularly Kupffer cells and macrophages, can express hedgehog ligands (Hh),^23^ which has been demonstrated in BA previously.^24^ Hh-stimulated cholangiocytes produce cytokines, including TGF-β.^25^ Both increased Hh pathway activity and TGF-β have been shown to stimulate EMT.^3,24^ TGF-β triggers destabilisation of adherens junctions, so β-catenin accumulates in cell nuclei and feeds Wnt signalling, which, as discussed, is involved in EMT and fibrosis in BA.^3^

The authors performed in vitro studies to investigate whether SULT2B1 was implicated in this process. Human intrahepatic biliary epithelial cells (HIBEpiC) were treated with TGF-β1, which led to increased protein expression of SULT2B1, MMP7 and mesenchymal markers. Markers of the Wnt/β-catenin pathway were also increased. Functional analysis of RNA-Seq data for these cells suggested EMT was activated after TGF-β1. This implies that SULT2B1 is involved in the cholangiocyte EMT process and is associated with Wnt/β-catenin activation. Further evidence for this was found by knocking down SULT2B1 gene expression in a HIBEpiC cell line. This led to lower SULT2B1 mRNA and protein expression levels as expected, as well as lower expression of Β-catenin, MMP7 and other EMT-related proteins. RNA-Seq functional analysis suggested SULT2B1 silencing inhibited the TGF-β1 signalling pathway and EMT. The authors therefore concluded that SULT2B1 promotes cholangiocyte EMT via Wnt/β-catenin and MMP7, and suggested that SULT2B1 acts as a downstream molecule of TGF-β.

This study cannot be immediately translated to clinical practice but suggests a mechanism which drives EMT and provides a possible therapeutic target for blockade in SULT2B1, which could theoretically become a disease-modifying therapy for BA. An important caveat to this is that while there is evidence that EMT and fibrosis are linked in BA, it is unclear how significant a driver EMT is as compared to the many other factors also contributing to fibrosis in BA.^26^ It is therefore uncertain how beneficial it would be to reduce EMT. Further investigation would also be needed to assess whether blockade of SULT2B1 is possible without prohibitive adverse effects, for example, affecting cholesterol metabolism.

Yang et al. also compared RNA-Seq results in patients who were CMV positive or negative from their cohort of sampled patients with BA, finding that SULT2B1 expression was significantly higher in the CMV positive vs. CMV negative group (*p* = <0.0001). MMP7 was also significantly overexpressed in the CMV-positive group (*p* = 0.0332). CMV is thought to be a contributor to the pathogenesis of BA, although it is uncertain whether it is a causative or exacerbating agent.^27,28^ Previous studies have reported that viruses may induce EMT in cholangiocytes and have hypothesised that this could be a survival response to minimise virus-induced apoptosis.^26,29^ This has previously been found to persist even after clearance of the virus.^29^ The evidence for a difference between the SULT2B1 and MMP7 expression in BA patients with CMV suggests another potential avenue for further research, as this may provide an explanation for the importance of CMV in BA. If sufficient evidence is found, this has potential for translation to clinical research and practice as safe treatment for CMV already exists.

This study also provides further evidence for the utility of MMP7 as a biomarker. MMP7 analysis found that serum MMP7 was increased in BA patients at the time of Kasai. This was positively correlated with higher gamma-glutamyl transferase (GGT), total bile acids (TBA), and liver stiffness on elastography. Twenty of 31 BA patients also had serum MMP7 analysis 3–6 months post-operatively, showing ongoing raised MMP7 and positive correlation with liver stiffness as well as other serum markers, including GGT, TBA and alanine aminotransferase. This is in keeping with previous studies showing MMP7 is elevated in BA,^12–20^ and reinforces its potential as a prognostic indicator, as it was positively correlated with clinical markers of disease severity. This could be helpful for patients and families as well as supporting clinical decision-making, helping clinicians to risk-stratify patients, and potentially to sub-group patients who could be targeted for monitoring and interventions, as well as for further clinical research. In contexts where families must decide between cadaveric or living-related organ donation, it could also potentially support families’ decision-making. However, there are possible practical constraints, including cost and access to MMP7 analysis. There has been much experience in BA of biomarkers which have not ultimately been widely adopted in clinical practice, as discussed in a recent Pediatric Research article.^30^ This includes serum bile acids,^31^ hyaluronic acid,^32–35^ and aspartate aminotransferase to platelet ratio index scoring.^36^

As well as having potential as a biomarker, MMP7 is another possible therapeutic target. Yang et al. hypothesise that MMP7 may be part of a positive feedback loop leading to increasing fibrosis, in which TGF-β1-induced SULT2B1 upregulates MMP7, and MMP7, in turn, further activates TGF-β1, amplifying cholangiocyte EMT and fibrosis, so blockade could potentially be beneficial. MMP7 has also been studied in pulmonary and renal fibrosis, and MMP7 gene knockout mice have been shown to have reduced progression of renal fibrosis.^37^ Pharmacological MMP7 blockade also reduced markers of renal fibrosis in mice.^37^

In conclusion, the extensive work of Yang et al. in this study has provided further insight into mechanisms of EMT in BA and has found evidence that SULT2B1 promotes EMT via the Wnt/β-catenin pathway and MMP7. Further work is needed to translate these findings to clinical practice, and there are multiple possible routes for this, including further investigation of the potential for SULT2B1 blockade, the use of MMP7 as a prognostic marker or as a therapeutic target, and the relationship between SULT2B1 and CMV. We believe this is a step forward towards a giant leap in our understanding of the pathogenesis of BA.
